# Cultivation modes impacting root microbiomes and metabolites in medicinal orchid *Dendrobium denneanum*

**DOI:** 10.3389/frmbi.2023.1287336

**Published:** 2023-12-20

**Authors:** Lin Chen, Haiyan Ding, Xin Chen, Jiaojiao Wang, Yuan Hu, Hongping Chen, Youping Liu

**Affiliations:** ^1^School of Pharmacy, Chengdu University of Traditional Chinese Medicine, Chengdu, China; ^2^Chengdu University of Traditional Chinese Medicine, State Key Laboratory of Southwestern Chinese Medicine Resources, Chengdu, China

**Keywords:** *Dendrobium denneanum* Kerr, root microbiome, metabolites, soil property, cultivation mode

## Abstract

**Introduction:**

The plant microbiome is the second genome of plants and is important for plant growth and health. *Dendrobium* is an epiphytic herbal plant of the family Orchidaceae that is often found attached to tree trunks or rocks and exhibits different cultivation modes. Microbiological and metabolite studies of *Dendrobium denneanum* Kerr (*D*. *denneanum*) in different cultivation modes can reveal important relationships between *Dendrobium* spp., their microbiomes, and their pharmacological substances, which is important for sustainable agricultural development and human health, particularly in the study of medicinal plants.

**Methods:**

In this study, three cultivation modes, living tree epiphytic (LT), stone epiphytic (SE), and pot cultivation (PO) of *D. denneanum* in the same environment were selected, and the metabolites were using ultra-high performance liquid chromatography-quadrupole time-of-flight mass spectrometry (UPLC-Q-TOF-MS). Subsequently, differential metabolites were screened, the rhizosphere and root endosphere microorganisms were sequenced via high-throughput sequencing, and the 16S rRNA gene/ITS sequences were obtained.

**Results:**

The main microbial taxa in the rhizosphere and root endosphere of *D. denneanum* included bacteria belonging to Proteobacteria, Actinobacteria, and Actinobacteria, and the fungi Basidiomycota and Ascomycota, whose abundances varied in different cultivation modes. Soil properties affect the composition of *D. denneanum* metabolites and root microbiome, among which, soil total phosphorus (TP) and pH in particular are important factors for soil microorganisms. Studies of root microbial communities have shown that root endosphere fungi are similar to rhizosphere fungi with microbial enrichment occurring from the external environment to the internal structures. Root microbial communities and metabolites correlation analyses revealed significant correlations between rhizosphere microbes, as well as endophytes and metabolites. For example, the rhizosphere bacterium genus *Occallatibacter* and root endosphere fungus *Clonostachys* showed a significant negative correlation with the pharmacodynamic substance gigantol in *D. denneanum* (P<0.05).

**Conclusion:**

This study elucidates the effects of different cultivation modes on *D. denneanum* from the perspective of microorganisms and metabolites, and investigates the effects of root microorganisms on metabolites. The findings enhance the current understanding of root microorganisms in orchid plants and provide a theoretical basis for the cultivation of *Dendrobium* spp., represented here by *D. denneanum*.

## 1 Introduction

The microbiome is proposed as a combination of “microbe” and “biome,” and argued that the host and the environment are a holistic ecological component that encompasses the microbial community and its interactions with the environment (Berg et al., 2020). Plants are genotype-dominant, and environmental factors drive plant-microbe co-evolution toward the formation of a symbiotic and reciprocal functional body (holobiont) (Hassani et al., 2018). Under natural conditions, plant health is dependent on complex and dynamic plant-microbe interactions. The members of the plant microbiome play important roles in plant growth and development, metabolism, disease resistance, and stress tolerance through mutualism and competition and thus contribute to plant life history (Huang et al., 2018; Wei et al., 2019). Therefore, investigating plant microbiomes carries considerable importance.

Orchidaceae is one of the largest families of monocotyledonous plants, with an estimated 28,000 species widely distributed across a variety of terrestrial ecosystems from the tropics to subpolar regions (Chen and Chen, 2011). The growth of orchid plants cannot be separated from that of orchid mycorrhizal fungi (OMF), which help in seed germination and growth of orchids, and orchid plants in turn depend on OMF to complete their life history (McCormick et al., 2009; Selosse et al., 2017). Furthermore, the presence of beneficial bacteria in the rhizosphere promotes plant growth, helps control pathogens, and alleviates abiotic stresses, such as water deficit stress (Haque et al., 2020). Therefore, focusing on the root microbiome of orchids is important for understanding their physiological activities.

*Dendrobium*, the second-largest genus within the Orchidaceae family, encompasses over 1,000 species (Burke et al., 2008). Widely distributed across Asia, Australia, and Europe, *Dendrobium* plants are recognized for their distinctive performance for warm, humid, ventilated environments and are often found attached to tree trunks or stones (Schuiteman et al., 2007). *Dendrobium* has a long history of use in China as food or herbal medicine. In Chinese tradition, it can improve stomach function, promote fluid production, nourish yin, and alleviate dryness. Modern pharmacological studies have shown that *Dendrobium* has a variety of pharmacological effects, including anti-inflammatory, antibacterial, antioxidant, antitumor, immunomodulatory, blood pressure-lowering, and blood sugar regulatory effects (Schuiteman et al., 2007). *D. denneanum* mainly originates from south-central Sichuan, Taiwan, and southeastern to northwestern Yunnan, where it is used as a food, herbal medicine, or ornamental plant (Li et al., 2023).

Research on microbe-plant-metabolite regulatory networks has revealed that beneficial bacteria can regulate plant metabolites, thus enhancing crop quality. For example, *Bacillus* and *Paraburkholderia* can promote the synthesis of carotenoids, flavonoids, phenolics, and total anthocyanins in strawberries, thus enhancing their antioxidant capacity and increasing their food value (Rahman et al., 2018). Studies on *Glycyrrhiza uralensis* Fisch revealed that *Lysobacter* in the rhizosphere is closely associated with the synthesis of glycyrrhizic acid, which is closely related to gene CYP72A154 (Zhong et al., 2022). Additionally, studies on *Reynoutria japonica* Houtt showed that the rhizosphere microbes such as *Bacteroides*, *Acinetobacter*, *Erysipelatoclostridium*, and *Achromobacter* enhanced the accumulation of resveratrol (Zhang et al., 2020).

The plant root microbiome and soil environment are closely interconnected, and soil type has been demonstrated to be an important determinant of the *Arabidopsis thaliana* rhizosphere and endophytic fungal and bacterial communities (Bulgarelli et al., 2012; Lindahl et al., 2013; Urbina et al., 2018). In the present study, we collected *D. denneanum* from three cultivation modes to study their root microbiomes and metabolites. We also analyzed the soil properties of the three cultivation modes to provide new insights into the relationship between *Dendrobium* spp. and their root microbiomes. Our objectives were to investigate whether different soil types affect the microbial and metabolite compositions of *Dendrobium* spp. in the same environment, whether endophyte microbes are related to rhizosphere microbes, and the effects of root microorganisms on metabolites.

## 2 Materials and methods

### 2.1 Plant material and soil sampling

In this study, we collected *D. denneanum* from three cultivation modes in the same environment, namely epiphytic cultivation on living trees (LT), epiphytic cultivation on red sandstone (SE), and potted cultivation in small gravel (PO). The three cultivation modes are represented by three distinct soil types. The *D. denneanum* used in this study was collected on January 9th, 2023, which is the local harvest season of *D*. *denneanum*. Plants were collected in Sichuan, China (29°44′2″N, 103°21′24″E), Six points were randomly selected for sampling in each plot, and one plant was selected from each point, all of which were collected. We took the roots and stems of the same plant as the subject of our study (Zhu et al., 2022). The stems were dried in an oven at 50°C to a constant weight for the determination of their composition (Xiao et al., 2022). Metabolite test samples were procured by subjecting desiccated sample powder to an 80% methanol solution (20 mg/mL DW). The resulting mixture was then subjected to sonication in an ultrasonic water bath, employing 100 watts of power and maintaining a temperature of 25°C for 30 minutes (Wu et al., 2020). The roots of the plants were collected and used to determine the microbial species composition of the rhizosphere and root endosphere. The “R” and “E” in front of the cultivation pattern abbreviation stand for rhizosphere and root endosphere, respectively. The rhizosphere soil was collected by lifting the plant out of the soil, shaking it vigorously, and collecting the soil that remained adhered to the roots. The roots were gently brushed with a sterile brush, the rhizosphere soil was collected in sterile tubes (Zuo et al., 2021). The roots were placed in new sterile tubes, and sterile phosphate buffer was added and vortexed until the buffer was clarified. Root surfaces for endophyte microbial sequencing were sterilized and used to extract total root endophyte DNA for sequencing. The sterilization method was performed as described by (Li et al., 2021). The collected samples were rapidly frozen in liquid nitrogen and stored at 80°C until DNA extraction. A total of 36 samples (six replicates × three cultivation modes; 18 root samples and 18 rhizosphere soil samples) were collected.

### 2.2 Measurement of soil physical and chemical properties

In this study, soils from three cultivation modes were examined, with three experimental soil groups and three replicates for each group. The pH of soil suspension 1:2.5 (W/V) was measured using a pH meter, soil organic carbon (OC) content was determined by using the H_2_SO_4_-K_2_Cr_2_O_7_ oxidation method, soil total nitrogen (TN) was determined using the Semimicro Macro Kjeldahl method(Wenjun et al., 2014), soil total phosphorus(TP) was determined via acid-soluble molybdenum-antimony colorimetry (Liu et al., 2022), and total potassium (TK) was measured via flame photometry (Mishra et al., 2020).

### 2.3 Qualitative study of chemical constituents in D. *denneanum* based on UPLC-Q-TOF-MS technology

We used ultra-high performance liquid chromatography-quadrupole time-of-flight mass spectrometry (UPLC-Q-TOF-MS) to characterize the metabolites of *D. denneanum* and preliminarily compared their contents based on their relative peak areas. An AccucoreTM C18 column (100 mm ×3 mm,2.6 μm) was used for gradient elution of mobile phase acetonitrile solution (B)-0.1% aqueous acetic acid solution (A) (**Supplementary Table 1**), under the following conditions: column temperature, 25.0°C; flow rate, 0.2 mL/min; injection volume, 4 µL. The mass spectrometry conditions were as follows: electrospray ionization with positive and negative ion mode detection, spray voltage of 3.2 kV, ion source temperature of 350°C, sheath gas flow rate of 40 arb, the auxiliary gas flow rate of 15 arb, and ion transfer tube temperature of 300°C. The scanning mode was Full MS/dd-MS2, with a primary resolution of 70 000, secondary resolution of 17 500, scanning range of m/z 100 to 1 500, and collision energy gradients of 20, 40, and 60 eV.

### 2.4 Identification and screening of metabolites

We imported the collected raw data into Compound Discoverer 3.0, established the identification process of unknown compounds through its wizard settings and method templates, performed peak alignment and peak extraction on the raw data, and set the filtering parameters for the matching results as follows: peak area threshold of 80,000 and mass deviation of the primary and secondary levels of 5 ppm. The extracted molecular ion chromatographic peaks and isotopic peaks were fitted to the possible molecular formulas, and the measured spectra of the secondary fragments were matched with the mzCloud and mzVaule network databases. A match score ≥80 was desirable.

The filtered ions were compared with the compound information in the databases HMDB 5.0 (Wishart et al., 2022) and PubChem (Kim et al., 2021), as well as the relevant literature to analyze and identify the compounds. Based on the peak area of each chromatographic peak represented the relative content of the corresponding substance. According to the peak area of the chromatographic peaks, the identified compounds in each group were subjected to orthogonal partial least squares-discriminant analysis (OPLS-DA) using SIMCA 14.1 to screen compounds with a Variable Importance in the Projection (VIP)>1, and IBM SPSS 26.0 (Chicago, IL, USA) was used for statistical analysis to screen the differential compounds with P<0.05.

### 2.5 DNA extraction, sequencing, and processing

Genomic DNA within soil and root samples was extracted using CTAB and the extracted genomic DNA was analyzed using 1% agarose gel electrophoresis. The primers ITS1-1F-F CTTGGTCATTTAGATACCCKG and ITS1-1F-R GCTGCGTTCTTCATCGATGC were used to amplify the fungal ITS1 ribosomal RNA gene internal transcribed spacer (ITS) region (Ihrmark et al., 2012). Primers 799F-AACMGGATTAGATACCCKG and 1193R-ACGTCATCCCCACCTTCC were used to amplify the highly variable regions of the bacterial and archaeal 16S rRNA gene fragment from the region V5-V7 (Beckers et al., 2016). Polymerase chain reaction (PCR) was performed using Bio-Rad T100 gradient PCR. The total volume of the PCR reaction mixture was 30 μL (15 μL of Phusion® High-Fidelity PCR Master Mix with GC Buffer (New England Biolabs, Inc. USA), 1 μM of forward and reverse primers and approximately 10 ng of genomic DNA). The PCR was performed as follows: 98°C for 1 min, 30 cycles of 98°C for 10 s, 50°C for 30 s, and 72°C for 30 s, followed by a final extension at 72°C for 5 min. PCR products were purified using a Universal DNA Purification Kit (TianGen, Beijing. China), and sequencing was performed on an Illumina Novaseq6000 (Illumina, Inc., United States) at Beijing Allwegene Technology Co., Ltd (Thermo Fisher Scientific, Waltham, MA, USA).

After truncating the barcode and primer sequences of each sample, the reads of each sample were spliced using FLASH (v1.2.11) (Magoč and Salzberg, 2011), and the raw Tags obtained from splicing were spliced. The Qiime software (v1.9.1) was performed to quality control and filter the spliced sequences, and the clean tags were obtained after strict filtering(Whelan and Surette, 2017). The clean tag sequences were compared with the species annotation database through GitHub (Dozmorov, 2018) to detect chimeric sequences, which were then removed to obtain the final effective tags. The Uparse algorithm (USEARCH v7) (Edgar, 2013) was used to cluster all the effective tags of all the samples, and the sequences were clustered into operational taxonomic units (OTUs) by default with 97% identity. The OTU sequences were analyzed for species annotation using the Mothur method with the SSU rRNA database of SILVA138.1 (Quast et al., 2013) (with a threshold value of 0.8-1), and taxonomic information was obtained at each taxonomic level. The phylogenetic relationships of all OTU representative sequences were obtained by rapid multiple sequence comparison using MAFFT (v7.490) software (Katoh and Standley, 2013). Finally, the data of each sample were homogenized, with the least amount of data in the sample as the criterion for rarefaction. Subsequent alpha diversity analysis and beta diversity analyses were based on the homogenized data and were conducted using the QIIME software (v1.9.1).

### 2.6 Statistical analysis

The Variable Importance in the Projection (VIP) values of the metabolites were extracted from the OPLS-DA results using SMICA 14.1 (Umetrics). Compounds with VIP values greater than 1 were screened and plotted for metabolite principal component analysis (PCA) using the OriginLab 2021 software (OriginLab Corporation, USA, 2021). Significant differences in metabolites were analyzed using a one-way analysis of variance performed using SPSS 26.0. Statistical analyses were performed using R version 4.1.2. The alpha diversity index and beta diversity distance matrices were calculated based on the OTUs and their abundance results using QIIME (version 1.9.1) software. Unifrac distances were calculated, Unweighted Pair Group Method with Arithmetic Mean (UPGMA) sample clustering trees were constructed, Principal coordinates analysis (PCoA) analytical plots were plotted using the ggplot2 package of R software, and the significant differences in the alpha diversity index were analyzed by SPSS 26.0. The beta diversity analysis was employed to evaluate the variation in root microbial communities across different cultivated substrates, utilizing the UniFrac distance measure. The resulting dissimilarities or similarities in the composition of the sampled communities were then visualized through PCoA. Additionally, a phylogenetic tree was constructed using the UPGMA method to elucidate and compare the relationships among the samples (Han et al., 2019). Venn diagrams were generated using the Venn diagram program (Chen and Boutros, 2011), and bar graphs were generated using Chiplot (https://www.chiplot.online/) based on the results of taxonomic annotation and relative abundance. The relationship between soil properties and rhizosphere bacterial-fungal community composition and diversity was investigated by redundancy analysis (RDA) using Canoco 5 software. Significant differences in soil properties were analyzed using SPSS 26.0. PICRUSt2 and FunGuild were used to analyze the rhizosphere and root endosphere bacteria for functional prediction (Nguyen et al., 2016; Douglas et al., 2020).

Linear Discriminant Analysis Effect Size (LEfSe) analyses were performed using LEfSe software with the default setting of an LDA Score filter value of 4. Combined metabolome and microbiome analyses were performed using the LEfSe method to analyze statistically different biomarkers. Correlations between fungi and bacteria screened at the genus level and coreference metabolites were analyzed using Pearson’s analysis, and the results are represented as heatmaps. Pearson’s correlation analyses were performed using SPSS 26.0. Chiplot was used for data visualization to generate bar charts, heat maps, and box plots.

## 3 Results

### 3.1 Physicochemical properties of rhizosphere soil in different cultivation modes

The physicochemical properties of the soil in the three cultivation modes are detailed in **Table 1**. There were no discernible disparities in the OC levels across the SE, LT, and PO groups. Similarly, the pH levels of the soil were found to be consistent among the three groups. However, notable discrepancies were observed in the concentrations of TP and TK between the SE, LT, and PO groups. Specifically, the LT group exhibited significantly higher levels of TN compared to both the PO and SE groups. Conversely, the TK levels in the LT group were significantly lower than those in the PO and SE groups (**Table 1**).

**Table 1:** Soil properties of different cultivation patterns.

Cultivation mode	pH	OC(g/Kg)	TN(mg/kg)	TP(mg/Kg)	TK(g/Kg)
SE	6.6 ± 0.07ab	84.3 ± 0.50ab	30.33 ± 1.62b	287.74 ± 13.17a	19.16 ± 0.46b
LT	4.73 ± 0.10b	86.75 ± 0.97b	434 ± 37.04a	211.23 ± 3.24b	5.78 ± 0.03c
PO	6.82 ± 0.12a	80.17 ± 0.41a	14.93 ± 1.62b	166.23 ± 3.82c	37.43 ± 1.52a

After the Tukey test, different letters after the data indicate statistically significant differences (p < 0.05).

### 3.2 Metabolites of *D. denneanum* in different cultivation modes

**Figure 1:**
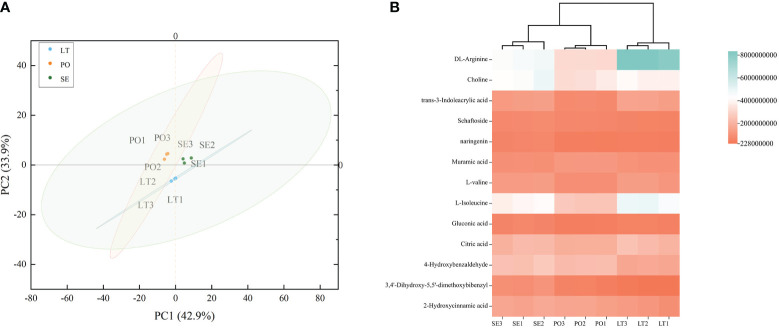
**(A)** Principal component analysis (PCA) plot showing different metabolites in different cultivation modes. **(B)** Clustered heat map to characterize metabolite peak areas for differences in content across cultivation modes. LT, SE and PO represented three cultivation modes: living tree epiphytic (LT), stone epiphytic (SE), and pot cultivation (PO).

UPLC-Q-TOF-MS was performed to investigate whether the metabolites of *D. denneanum* differed among the different cultivation modes. The Ion flow diagram of metabolites of *D. denneanum* is presented in **Supplementary Figure 1**. A comparison of the mass spectrometry data, revealed 64 compounds, and the results are shown in **Supplementary Table 2**. The explanation by variable X (PC1) was 42.9%, whereas that by variable Y (PC2) was 33.9% when the samples of each group were analyzed for differences (**Figure 1A**). Screening for differential metabolites (VIP>1, P<0.05) in each cultivation pattern, as shown in **Table 2**, revealed a total of 13 differential metabolites, among which DL-arginine, 4-hydroxybenzaldehyde, L-isoleucine, and 3,4’-dihydroxy-5,5’-dimethoxybibenzyl (gigantol) were all significantly different (**Figure 1B**). Notably, gigantol, an important class of pharmacodynamic components in *D. denneanum*, is a selective inhibitor of nuclear transcription factor κB, which possesses a variety of pharmacological effects, including anti-inflammatory activity, amelioration of diabetes mellitus, and anti-tumor activity (Charoenrungruang et al., 2014; Wu et al., 2015; Xue et al., 2020), and is, therefore, worthy of further investigation.

**Table 2:** Differential metabolites in different cultivation modes.

Compounds	SEvsLT		SEvsPO		LTvsPO	
	VIP	typle	VIP	typle	VIP	typle
DL-Arginine	4.74	Up	3.29	Down	4.97	Down
4-Hydroxybenzaldehyde	2.26	Down	1.11	Down	1.74	Up
L-Isoleucine	2.2	Up	3.22	Down	3.28	Down
3,4'-Dihydroxy-5,5'-dimethoxybibenzyl	2.07	Down	1.79	Down	1.1	UP
Choline	1.77	Down	2.85	Down	−	−
2-Hydroxycinnamic acid	1.63	Up	−	−	1.32	Up
Schaftoside	1.1	Down	−	−	−	−
trans-3-Indoleacrylic acid	−	−	2.06	Down	1.84	Down
L-valine	−	−	1.77	Down	1.36	Down
Muramic acid	−	−	1.37	Up	−	−
naringenin	−	−	1.23	Down	−	−
Gluconic acid	−	−	1.15	Down	−	−
Citric acid	−	−	−	−	1.18	Down

### 3.3 Root microbiome community composition and alpha diversity in different cultivation modes

A comprehensive analysis was conducted on a total of 64 samples, including 16S rRNA and ITS amplicons. The sequencing effort yielded a substantial number of reads, with 2,300,515 16 rRNA gene reads assigned to bacterial communities and 2,869,398 reads designated for fungal communities. This resulted in an average of 80,780 reads per sample. The subsequent clustering process, based on the principle of 97% sequence similarity, generated a total of 9390 OTUs for rhizosphere bacteria, 2249 OTUs for rhizosphere fungi, 9568 OTUs for root bacteria, and 2150 OTUs for root fungi. Notably, the bacterial networks displayed a higher degree of complexity compared to the fungal communities.

**Figure 2:**
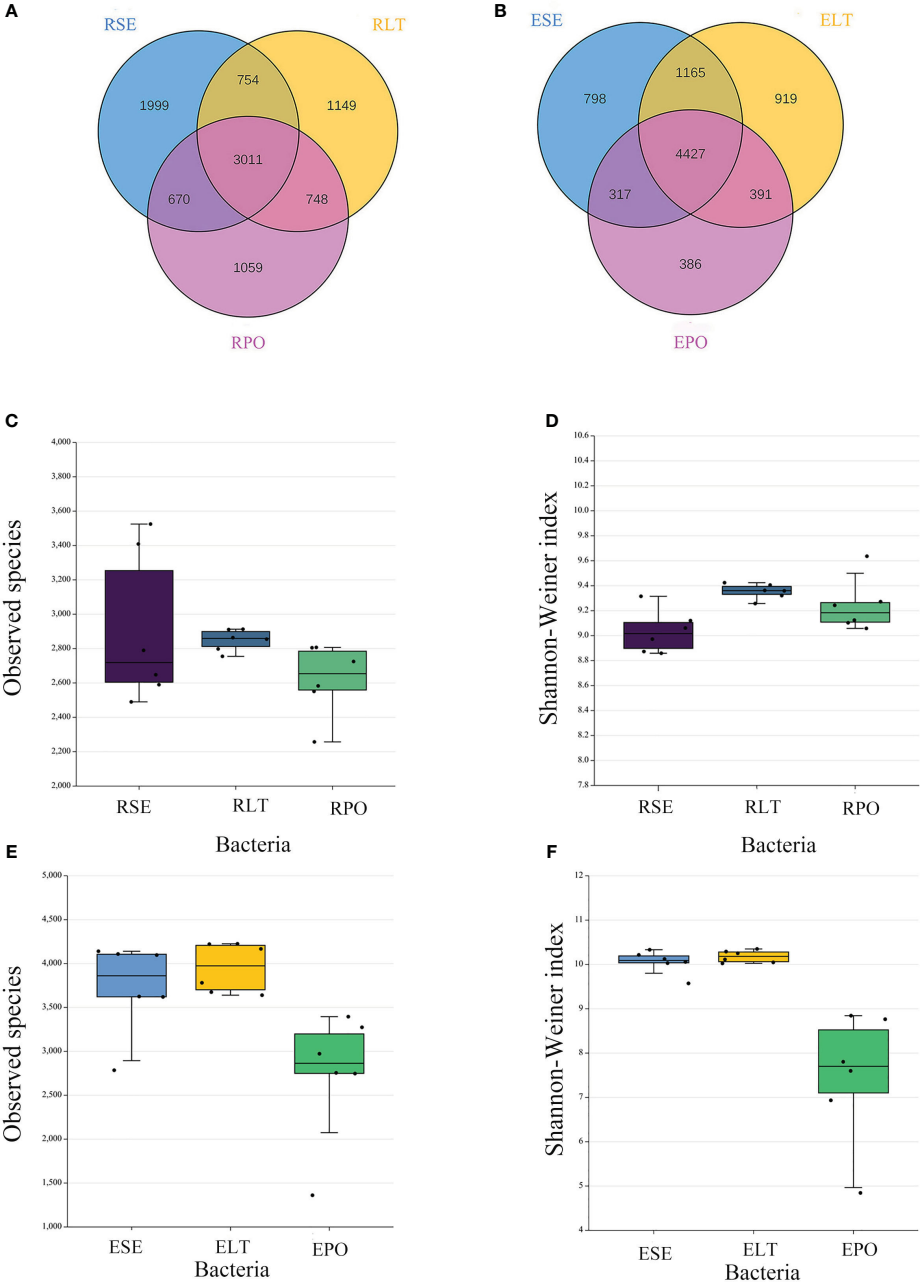
**(A, B)** Operational taxonomic unit (OTU)-based Venn diagram analysis of group-specific OTU numbers **(A)** for rhizosphere bacteria **(B)** for root endosphere bacteria, **(C–F)** Boxplot of observed species and Shannon–Weiner index exponents to characterize differences in alpha diversity **(C, D)** for rhizosphere bacteria **(E, F)** for root endosphere bacteria. The “R” and “E” in front of the cultivation pattern abbreviation stand for rhizosphere and root endosphere.

**Figure 3:**
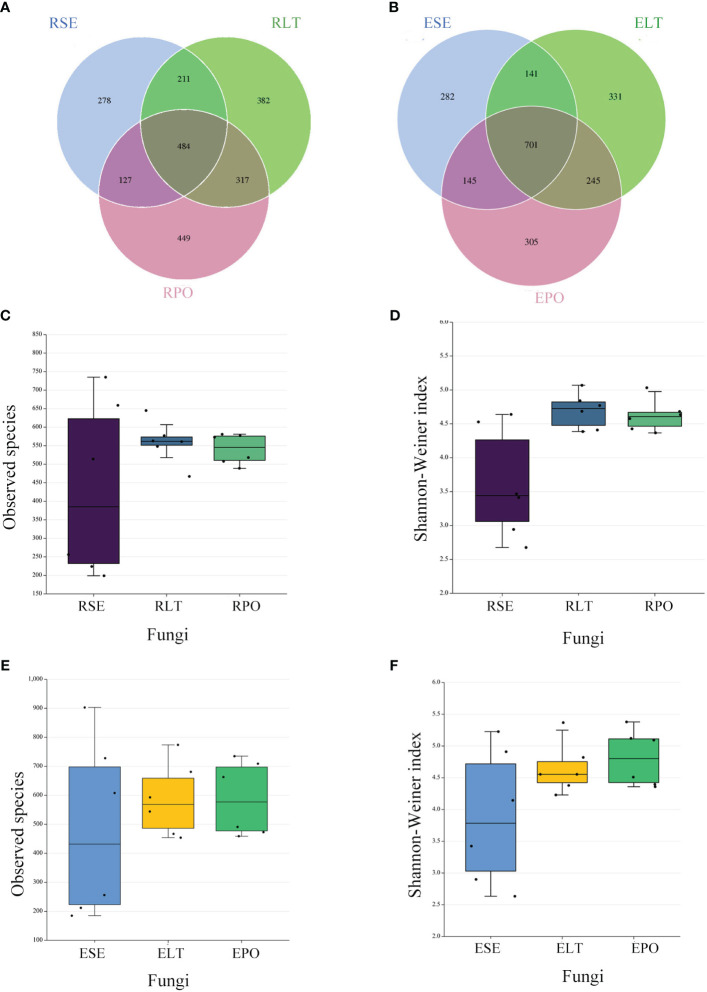
**(A, B)** Operational taxonomic unit (OTU)-based Venn diagram analysis of group-specific OTU numbers **(A)** for rhizosphere fungi **(B)** for root endosphere fungi, **(C–F)** Boxplot of observed species and Shannon–Weiner index exponents to characterize differences in alpha diversity **(C, D)** for rhizosphere fungi **(E, F)** for root endosphere fungi a. The “R” and “E” in front of the cultivation pattern abbreviation stand for rhizosphere and root endosphere.

Venn diagrams showed that the root microbiomes differed among the different cultivation modes, and the root microorganisms of each cultivation substrate had their unique communities. Among the rhizosphere microbes, SE had 1999 and 278 unique bacterial and fungal OTUs, respectively, LT had 1,149 and 382 unique bacterial and fungal OTUs, respectively, and PO had 1,059 and 449 unique bacterial and fungal OTUs, respectively. Among the root endosphere microbes, SE possessed 798 and 282 unique bacterial and fungal OTUS, respectively, LT possessed 919 and 331 unique bacterial and fungal OTUs, respectively, and PO possessed 386 and 305 unique bacterial and fungal OTUs, respectively (**Figures 2A, B**, **3A, B**).

The α-diversity is utilized to analyze the diversity of microbial communities within a given ecosystem, reflecting the richness and variety of microbial species within the sampled population (**Supplementary Table 3**). Through the utilization of observed species and Shannon-Weiner indices, it has been determined that, in both rhizosphere and endophytic microbiota, the bacterial α-diversity within the PO group exhibited a significantly diminished state compared to the SE and LT groups (P<0.05). However, no discernible disparities were detected among the three groups in terms of the α-diversity of both endophytic and rhizosphere fungi (**Figures 2C–F**, **3C–F**).

### 3.4 Composition of the bacterial community in different cultivation modes

**Figure 4:**
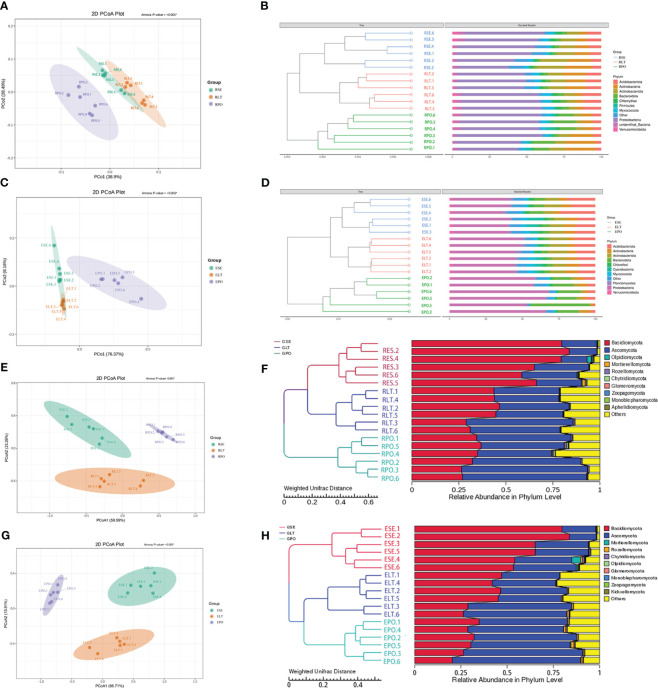
**(A, C, E, G)** PCoA based on Weighted Unifrac distance at the OTU level, **(B, D, F, H)** unweighted pair group method with arithmetic mean (UPGMA) cluster analysis based on Weighted Unifrac distance matrix. The closer the sample is, the shorter the length of the branches, showing that the species composition of the two ecotypes is similar. **(A, B)** rhizosphere bacteria, **(C, D)** root endosphere bacteria, **(E, F)** rhizosphere fungi, **(G, H)** root endosphere fungi.

**Figure 5:**
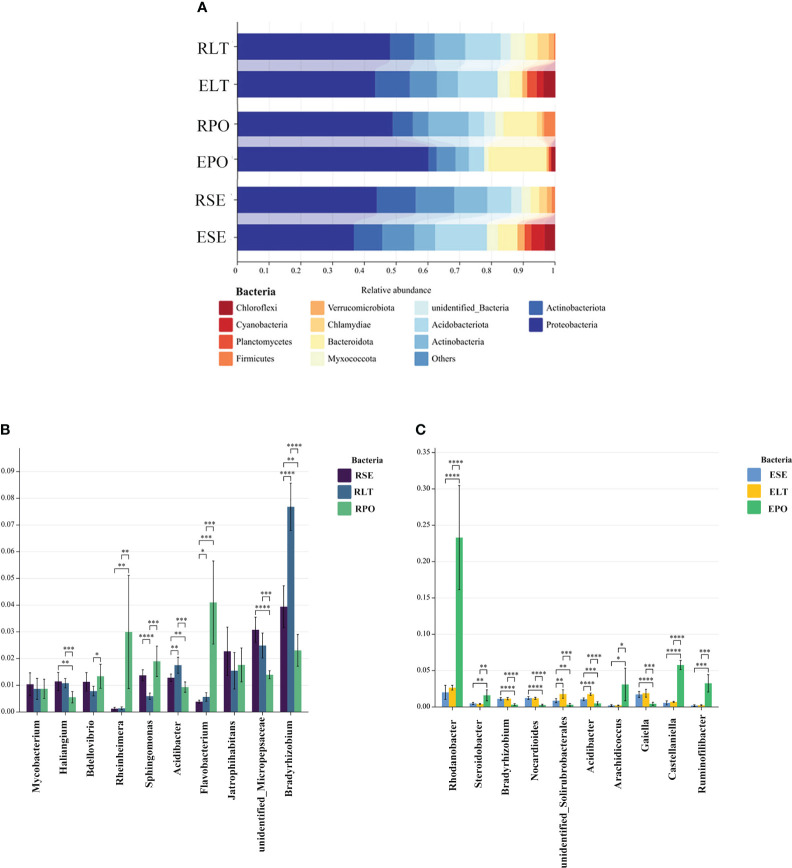
**(A)** Show the relative abundances of rhizosphere and root endosphere bacterial communities at the phylum level in different cultivation modes. **(B, C)** Differences between cultivation modes at the genus level. The x-axis represents the genus levels of species, and the y-axis represents the percentage of species average relative abundance in each sample group. **(B)** rhizosphere bacteria, **(C)** root endosphere bacteria. (*:0.01<P<0.05; **:0.001<P<0.01; ***:0.001<P<0.0001; ****:P<0.0001.

As depicted in **Figure 4**, the findings unveiled a coherent clustering of the six biological replicates, both in terms of inter-root fungi and bacteria, as well as intra-root fungi and bacteria. Notably, the diversity exhibited significant variation across the different cultivation modes, thereby implying the influence of the specific cultivation pattern on the root microbial community. In the composition of bacterial communities, Proteobacteria, Actinobacteriota, and Actinobacteria were the dominant bacterial phyla in rhizosphere and root endosphere. The abundance of Proteobacteria was higher in PO than in SE and LT, whereas the abundances of Actinobacteriota and Actinobacteria were less abundant than those in SE and LT. To highlight the effect of cultivation mode on the root microbiome, we examined the relative abundance of the top ten genera among the rhizosphere and root endosphere bacteria in different cultivation modes. Among the rhizosphere bacteria *Haliangium*, *Rheinheimera*, *Sphingomonas*, *Acidibacter*, *Flavobacterium*, *unidentified_Micropepsaceae*, and *Bradyrhizobium* differed significantly among the three cultivation modes (P< 0.05). *Bradyrhizobium* had the highest abundance in LT, *unidentified_Micropepsaceae* had the highest abundance in SE, and *Flavobacterium* had the highest abundance in PO. Among the root endophytes, *Steroidobacter*, *Bradyrhizobium*, *Nocardioides*, *unidentified_Solirubrobacterales*, *Acidibacter*, *Arachidicoccus*, *Gaiella*, *Castellaniella*, *Ruminofilibacter*, and *Rhodanobacter* differed significantly among the three cultivation modes (P<0.05), with *Steroidobacter* having the lowest abundance in LT. In summary, it can be stated that different cultivation modes exert an influence on the composition of bacterial communities, both in the rhizosphere and the endosphere (**Figure 5**).

### 3.5 Composition of the fungal community in different cultivation modes

**Figure 6:**
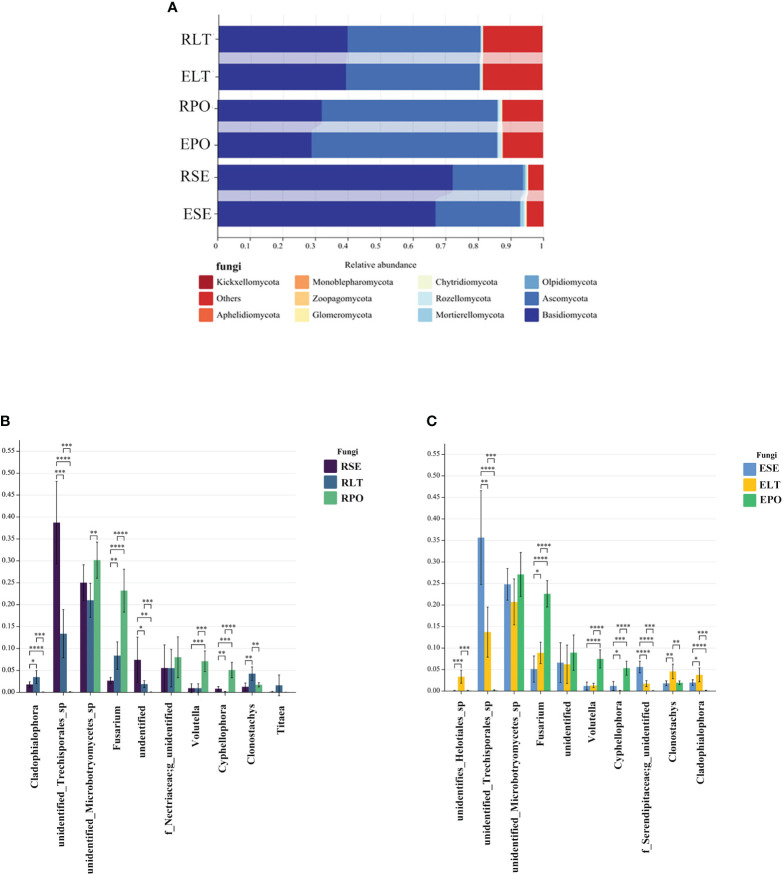
**(A)** Show the relative abundances of rhizosphere and root endosphere fungal communities at the phylum level in different cultivation modes. **(B, C)** Differences between cultivation modes at the genus level. The x-axis represents the genus levels of species, and the y-axis represents the percentage of species average relative abundance in each sample group. **(B)** rhizosphere fungi, **(C)** root endosphere fungi. (*:0.01<P<0.05; **:0.001<P<0.01; ***:0.001<P<0.0001; ****:P<0.0001.

Basidiomycota and Ascomycota were the dominant fungal phyla in both the rhizosphere and the root endosphere, the abundance of Basidiomycota was significantly higher in the SE group compared to the LT and PO groups, while the abundance of Ascomycota was comparatively lower than that of the LT and PO groups. In terms of root endosphere fungi, unidentified *Trechisporales*, *Fusarium*, *Volutella*, *Cyphellophora*, *Clonostachys*, and *Cladophialophora* differed significantly (P<0.05) among the three cultivation modes, with unidentified *Trechisporales* having the highest abundance in SE, and unidentified *Microbotryomycetes* having the highest abundance in LT and PO. Among the rhizosphere fungi, unidentified *Trechisporales*, *Fusarium*, *Volutella*, *Cyphellophora*, *f*_*Serendipitaceae, g*_unidentified, *Clonostachys*, *Cladophialophora*, and unidentified *Helotiales* differed significantly among the three cultivation modes (P<0.05) unidentified *Trechisporales sp* had the highest abundance in SE, and unidentified *Microbotryomycetes sp* in LT and PO had the highest abundance, which was consistent with rhizosphere fungi (**Figure 6**).

**Figure 7:**
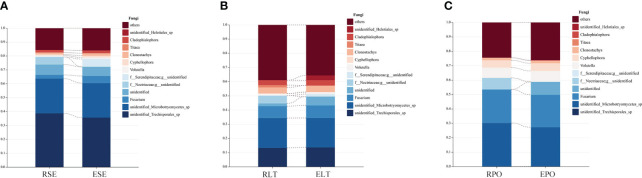
**(A–C)** show dominant genera and percentage of rhizosphere and root endosphere fungi in different cultivation modes and connect the same genera by dotted line. **(A)** Rhizosphere and root endosphere fungi in SE. **(B)** Rhizosphere and root endosphere fungi in LT. **(C)** Rhizosphere and root endosphere fungi in PO.

We compared the root-endosphere fungi with the rhizosphere fungi in the topen genera of relative abundance at the genus level in different cultivation modes and found that although the top ten genera of relative abundance at the genus level were the same in different cultivation modes, the abundance varied significantly among genera, and the composition of the root-endosphere fungal community was similar to that of the rhizosphere fungal community in each cultivation mode. Among them, unidentified *Trechisporales* and unidentified *Microbotryomycetes* cultivated in SE were the dominant genera, and unidentified *Trechisporales* accounted for 38.72% in the rhizosphere microbiome of *D. denneanum* in the epiphytic habitat of living stones (RSE) and 35.66% in the root endosphere microbiome of *D. denneanum* in the epiphytic habitat of living stones (ESE), while unidentified *Microbotryomycetes* accounted for 25.02% of RSE and 24.81% of ESE. 35.66% of RSE, *unidentified Microbotryomycetes* 25.02% in RSE and 24.81% of ESE; LT cultivated unidentified *Trechisporales*, unidentified *Microbotryomycetes*, and unidentified *Microbotryomycetes* were the dominant genera.unidentified *Trechisporales sp* accounted for 13.37% in the rhizosphere microbiome of *D. denneanum* in the epiphytic habitat of living trees (RLT) and 13.71% in the root endosphere microbiome of *D. denneanum* in the epiphytic habitat of living trees (ELT); unidentified *Microbotryomycetes* accounted for 21.00% in RLT and 20.71% in ELT; unidentified *Microbotryomycetes* and *Fusarium* were the dominant genera in PO, unidentified *Microbotryomycetes* in the rhizosphere microbiome of *D. denneanum* in the pot cultivation (RPO) 30.16%, in ELT 27.10%. *Fusarium* is 23.20% in RPO and 22.59% in ELT. Our study showed the variation in the root microbiome of *D. denneanum* due to distinct cultivation modes and soil types. Additionally, when comparing the fungal community of the *D. denneanum* root endosphere with that of the rhizosphere, a closer similarity was observed, thereby suggesting the influence of rhizosphere fungi on the root endosphere fungi (**Figure 7**).

### 3.6 Prediction of the function of the *D. denneanum* root microbiome in different cultivation modes

**Figure 8:**
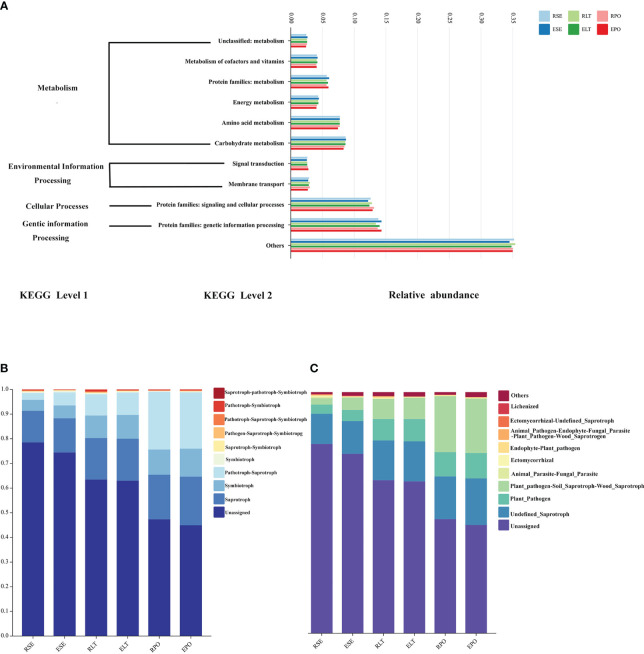
**(A)** Bar graphs for predicting bacterial community function. **(B)** Bar graphs for predicting fungal community function in Trophic Mode. **(C)** Bar graphs for predicting fungal community function in Guild.

We employed PICRUSt2 to scrutinize the bacterial communities residing within the rhizosphere and root endosphere, under distinct cultivation modes. We aimed to predict the functional attributes of these communities, drawing upon the comprehensive Kyoto Encyclopedia of Genes and Genomes database (KEGG) (Douglas et al., 2020). At level 1, metabolism, environmental information processing, cellular processes, and genetic information processing were included. Included at level 2 were the unclassified: metabolism, metabolism of cofactors and vitamins, protein families: metabolism, energy metabolism, amino acid metabolism, and carbohydrate metabolism attributed to metabolism; signal transduction and membrane transporter attributed to environmental information processing; protein families: signal transduction and cellular processes attributed to cellular processes; and protein families: genetic information processing attributed to genetic information processing. The results of our analyses showed no significant differences in the abundance of any of these pathways in the rhizosphere and root endosphere under different cultivation modes (**Figure 8A**).

Furthermore, we used FunGuild to predict the ecological functions of the rhizosphere and root endosphere fungi in different cultivation modes and statistically calculated the results of their functional abundance in both the Trophic Mode and Guild (**Figures 8B, C**) (Nguyen et al., 2016). In terms of Trophic Mode, the abundance of rhizosphere and root endosphere fungi in Pathotroph-Saprotroph and Pathotroph functions were significantly different (P<0.05) between SE and PO, and the abundance of rhizosphere and root endosphere fungi in Pathotroph-Saprotroph were significantly different between SE and LT, Pathotroph-Saprotroph, Pathotroph-Symbiotroph. Additionally, Pathotroph-Saprotroph functions were significantly different (P<0.05), and the abundance of rhizosphere and root endosphere fungi in Pathotroph-Saprotroph, Pathotroph-Saprotroph and Pathotroph-Symbiotroph functions in the SE was significantly different (P<0.05) from that in the PO. In the Guild, the abundance of Plant_Pathogen-Soil_Saprotroph-Wood_Saprotroph function was significantly (P<0.05) different among SE, LT, and PO treatments, and the abundance of Plant_Pathogen function was significantly (P<0.05) different among the SE *vs*. LT and SE *vs*. PO. The abundance of these functions was significantly different (P<0.05). The results showed that different microbial ecological functions were mostly pathogenic, suggesting that the selection of appropriate cultivation modes plays a role in protecting against plant diseases. Our results suggest that the SE of rhizosphere microorganisms possesses fewer pathogenic microbes.

### 3.7 Influence of soil properties on the composition and structure of rhizosphere microbes

**Figure 9:**
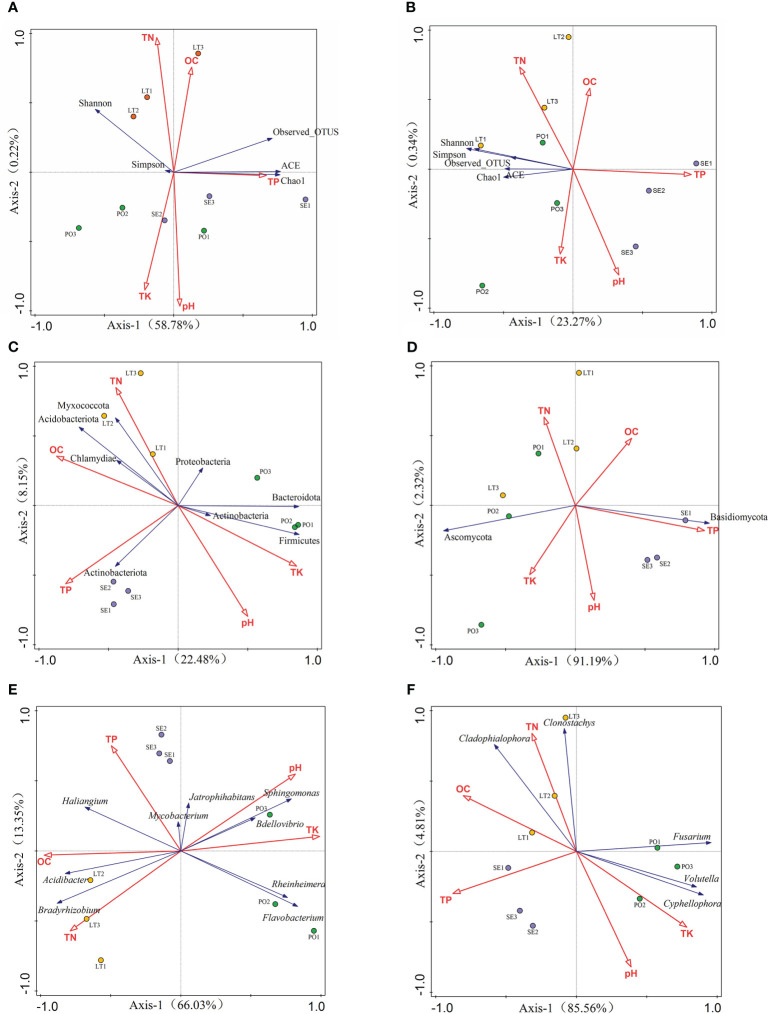
**(A, B)** Redundancy analysis (RDA)of soil properties and inter-root microbial α-diversity. **(C, D)** RDA of soil properties and rhizosphere microorganisms with >1% abundance at the phylum level. **(E, F)** RDA of soil properties and rhizosphere inter-root microorganisms with >1% abundance at the genus level **(A, C, E)** for rhizosphere bacteria. **(B, D, F)** represents rhizosphere fungi.

To better understand the relationship between microbial communities and soil types in different cultivation modes, we examined the soil physicochemical properties in the three cultivation modes. Rhizosphere bacterial diversity, rhizosphere fungal diversity, and soil properties were subjected to a detrended correspondence analysis (DCA), and the results showed that the maximum gradient lengths were less than 3. RDA was used to investigate the relationship between bacterial diversity and soil properties and the relationship between fungal diversity and soil properties (**Figure 9**). The results showed that soil properties contributed 59% of the total eigenvalues of rhizosphere bacterial diversity. The eigenvalues of the first two binning axes of RDA explained 58.78% and 0.22% of the variance in rhizosphere bacterial community diversity, respectively. Among them, TP (P=0.024) and pH (P=0.014) had a significant effect on the variance in rhizosphere bacterial community diversity and explained 26.2% and 21.5% of the variations in diversity, respectively. TN and OC were positively correlated with all five indices; TP content was positively correlated only with the ACE, Chao1, and Observes_OTUS indices; pH was positively correlated with Simpson, ACE, and Chao1 indices, TK content was positively correlated with the Simpson index, and the remaining soil properties were positively correlated with the index of alpha diversity. The remaining soil properties and the alpha diversity index were negatively affected. Soil properties contributed 33.1% to the total eigenvalues of alpha diversity of rhizosphere fungi, and the eigenvalues of the first two sorting axes of RDA explained 23.27% and 0.34% of the variance in the diversity of the rhizosphere fungal communities, respectively TN and TK were positively correlated with the five indices, and OC, TP, and pH were negatively correlated with the five indices.

DCA results showed that the maximum gradient length was less than 3 for communities and soil properties with greater than 1% abundance of rhizosphere bacteria as well as rhizosphere fungal phyla and genera. At the rhizosphere bacterial phyla level, RDA showed that soil properties contributed 33.1% to the total eigenvalues. At the rhizosphere bacterial genera level, the RDA showed that soil properties contributed 81.1% to the total eigenvalues and that TK (P=0.002) and pH (P=0.002) had a significant effect on the abundance of rhizosphere bacterial genera. At the rhizosphere fungal phylum level, RDA showed that soil properties contributed 93.5% of the total eigenvalues, and TK (P=0.002), pH (P=0.002), and OC (P=0.036) had a significant effect on the abundance of the rhizosphere fungal phyla, explaining 83.7%, 11.2%, and 2.6% of the variation in diversity, respectively. At the level of rhizosphere fungal genera, RDA showed that soil properties contributed 88.4% of the total eigenvalues, and TP (P=0.002) and TK (P=0.002) had a significant effect on rhizosphere fungal genera.

### 3.7 Microbial and metabolite correlation analysis

Microbes colonize in plant-associated microenvironments and play important roles in protecting and maintaining plant ecosystems (Mendes et al., 2013; Korenblum et al., 2020). Numerous studies have shown that microbes significantly affect the synthesis and accumulation of metabolites in their hosts (Schmidt et al., 2014; Arora et al., 2016; Maggini et al., 2017; Taghinasab and Jabaji, 2020). Analysis of microbial data has shown that different cultivation modes enrich different microbial communities of the root microbiome, which may have different functions. Therefore, we used LEfSe to analyze biomarkers in the root microorganisms and Pearson’s analysis of the correlation between these microbes and metabolites.

**Figure 10:**
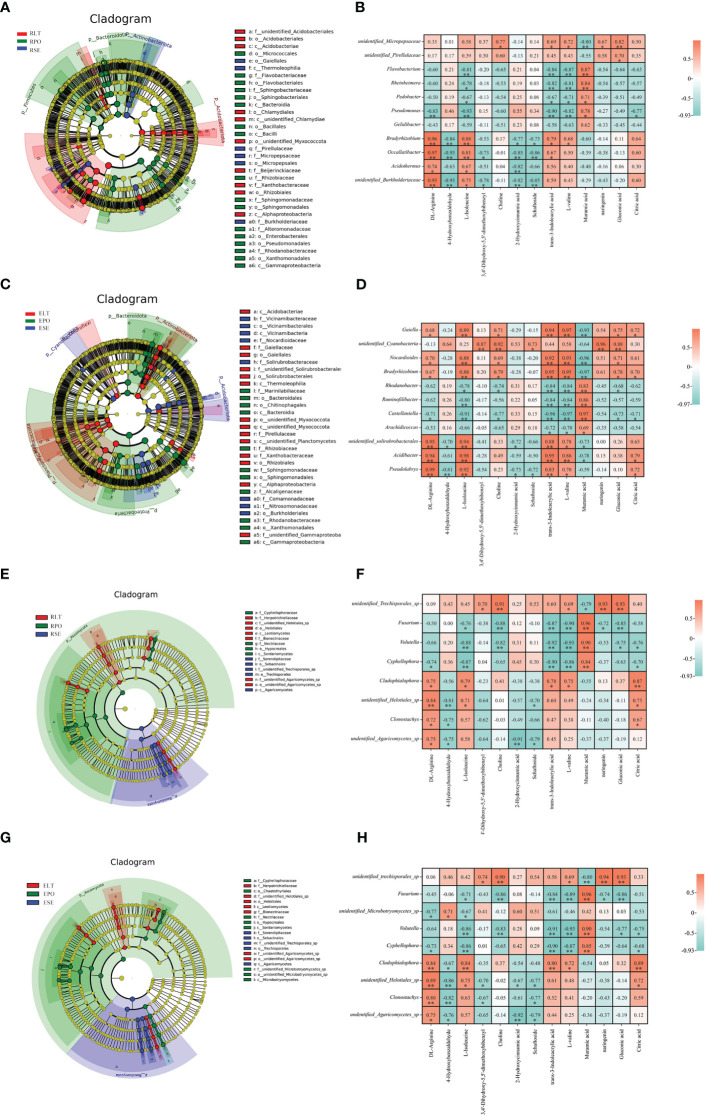
**(A, C, E, G)** Operational taxonomic unit (OTU) based evolutionary branching diagram (In an evolutionary branching diagram, circles radiating from inside to outside represent taxonomic levels from phylum to genus (or species). Each small circle at a different taxonomic level represents a taxon at that level, and the size of the circle diameter is proportional to the size of the relative abundance). **(B, D, F, H)** correlation heatmap based on Pearson correlation analysis to reveal microbial-metabolite correlations(*:0.01<P<0.05; **: P<0.01). **(A)** Branching diagram of rhizosphere bacteria. **(B)** Correlation heatmap between rhizosphere bacteria and metabolites. **(C)** Branching diagram of root endosphere bacteria. **(D)** Correlation heatmap between root endosphere bacteria and metabolites. **(E)** Branching diagram of the root rhizosphere fungi. **(F)** Correlation heatmap of rhizosphere fungi and metabolites. **(G)** Branching diagram of the root s root endosphere fungi. **(H)** Correlation heatmap of the root endosphere fungi and metabolites.

The LEfSe results revealed statistically differences in microbial communities in SE, LT, and PO cultures. LDA values greater than 4 were screened for biomarkers (**Supplementary Figure 2**). At the genus level, the rhizosphere bacterial biomarkers were unidentified *Micropepsaceae* and unidentified *Pirellulaceae* for SE, *Bradyrhizobium*, *Occallatibacter*, *Acidothermus* and unidentified *Burkholderiaceae* for LT, and *Flavobacterium*, *Rheinheimera*, *Pedobacter*, *Pseudomonas*, and *Gelidibacter* for PO. The rhizosphere fungal biomarkers in SE were unidentified *Trechisporales*, those in LT were *Cladophialophora*, unidentified *Helotiales*, *Clonostachys*, and undentified *Agaricomycetes*, and those in PO were *Fusarium*, *Volutella* and *Cypripedium*. The bacterial biomarkers in the root endosphere of SE were *Gaiella*, unidentified *Cyanobacteria*, *Nocardioides*, and *Bradyrhizobium*, those in LT were unidentified *Solirubrobacterales*, *Acidibacter*, and *Pseudolabrys*, and those in PO were *Rhodanobacter*, *Ruminofilibacter*, *Castellaniella* and *Arachidicoccus*. In the root endosphere of each cultivation model, fungal biomarkers were consistent with the rhizosphere fungal biomarkers, except for an additional unidentified *Microbotryomycetes* in PO, which may be related to the similarity between the root endosphere and rhizosphere fungal communities (**Figure 10**).

We used Pearson correlation analysis to investigate the rhizosphere and root endosphere bacterial and fungal biomarkers at the genus level and to identify differential metabolites. We screened for microbial-metabolite correlations greater than 0.95, with positive correlations at the rhizosphere level between unidentified *Cyanobacteria* and naringenin, negative correlations between *Nocardioides* and muramic acid, positive correlations between *Bradyrhizobium* and trans-3-indoleacrylic acid as correlations between L-valine and Muramic acid. *Castellaniella* was negatively correlated with trans-3-indoleacrylic acid as well as with L-valine and positively correlated with muramic acid. *Acidibacter* was positively correlated with L-isoleucine and trans-3-indoleacrylic acid. At the root endosphere level, *Bradyrhizobium* and *Occallatibacter* were positively correlated with DL-arginine. At both the rhizosphere and root endosphere levels, *Fusarium* was positively correlated with muramic acid, which may be related to the similarity of the fungal community within the root endosphere to the rhizosphere fungal community. Our study suggests that microbes are associated with plant metabolites.

## 4 Discussion

### 4.1 Soil properties influence the composition of microbial communities in the rhizosphere of *D*. *denneanum*, with the presence of co-dominant flora in *Dendrobium* spp.

Soil is the main biobank of terrestrial ecosystems, and plants interact with the soil through the root system and take up rhizospheric microbial communities from the soil. Soil type is a key factor determining the composition of rhizospheric microbial communities (Sneha et al., 2021; Peng et al., 2023). In the present study, we found that different soil types affected the rhizosphere microbial composition of *D. denneanum* under the same conditions, supporting our first hypothesis. Investigation of the soil properties of the three cultivation modes revealed that TP and TK differed significantly among the three cultivation modes (P<0.05). Our analysis results indicated that soil TP and pH have important effects on soil microorganisms. Moreover, Cao et al. reported that soil pH strongly affects fungi and bacteria (Lauber et al., 2008; Cao et al., 2016; Finkel et al., 2019), and Finkel et al. reported that plant phosphorus starvation response drives the effect of soil phosphorus content on plant microbiota, consistent with the results of this study (Finkel et al., 2019). At the phylum level, Proteobacteria, Actinobacteria, and Actinobacteriota were the most abundant bacteria in the rhizosphere and root endosphere, while Basidiomycota and Ascomycota were the most abundant fungi in the rhizosphere and root endosphere consistent with the findings of the studies of *Dendrobium huoshanense* (Chen et al., 2020), *Dendrobium nobile* (Zhao et al., 2023), and *Dendrobium officinale* (Zhu et al., 2022). A common dominant bacterial flora may exist among *Dendrobium* spp. Beyond that, the abundance of microorganisms in different cultivation modes varied, with Actinobacteriota and Basidiomycota being more abundant in SE than in the other two cultivation modes, and Proteobacteria and Ascomycota being more abundant in PO than in the other two cultivation modes, demonstrating the effect of soil properties on microbes.

### 4.2 Rhizosphere and root endosphere fungal communities of *D. denneanum* are similar in composition and perform varying ecological functions in different cultivation modes

The external environment, including soil and airborne particles, is an important source of plant microbes (Lebeis, 2015). The roots serve as the locus of intricate plant-microbe interplays, where the release of chemical signals by the roots engenders the aggregation of particular soil microbes around them, giving rise to a rhizosphere microbiome. Within this milieu, the plant exerts its influence by selectively recruiting microbes to colonize its internal tissues, thereby establishing an endophyte microbiome. This symbiotic association substantially augments the overall fitness of the plant. (Reinhold-Hurek et al., 2015; Zhang et al., 2017). Our results show that rhizosphere and root endosphere fungi not only share the same dominant genera across cultivation patterns but that these genera also have similar abundances. The enrichment of external plant microbes supports the conclusions of this study (Zhang et al., 2017). Furthermore, mycorrhizal fungi are required during orchid growth to facilitate mineral uptake and carbon replenishment, and thus growth and development (Gebauer and Meyer, 2003; Stöckel et al., 2014). Our results provide a basis for determining the origins of mycorrhizal fungi in orchids.

Moreover, functional predictions unveiled distinctions in the ecological roles performed by fungal communities in different cultivation modes. Specifically, root microbial communities in SE cultivation exhibited a reduced presence of pathogenic microorganisms, thus offering valuable insights for the prospective agro-cultivation of *D. denneanum*.

### 4.3 Different cultivation modes affect the synthesis of metabolites in *D. denneanum*, a process associated with root microorganisms

Studies have unveiled that rhizosphere microorganisms possess the capacity to elicit the secretion of root metabolites, exerting an influence on the transcriptional and metabolic profiles of plants, thereby modulating the synthesis of plant metabolites (Korenblum et al., 2020). As observed in the research on *Catharanthus roseus*, it was found that *Staphylococcus sciuri* and *Micrococcus* sp. have the potential to enhance the biosynthesis of terpenoid indole alkaloids (Tiwari et al., 2013). Our study found differences in *D. denneanum* metabolites under different cultivation modes, suggesting that different cultivation modes affect metabolite synthesis. We observed a substantial variance in the gigantol content, a pharmacologically active compound in *D. denneanum*, across diverse cultivation methods. Specifically, the gigantol content in SE markedly surpassed that in PO, with the latter, in turn, significantly exceeding LT. This information may guide future crop cultivation. To investigate whether the synthesis of its metabolites is related to root microorganisms, we conducted a Pearson correlation analysis and found that both inter- and intra-root microorganisms may affect the synthesis of metabolites. *Occallatibacter* and *Clonostachys* are significantly associated with the synthesis of gigantol (P<0.05). This observation implies a clear association between microorganisms and plant metabolites and suggests that microorganisms are indeed associated with plant metabolites and that microorganisms may influence host metabolism through certain mechanisms, such as the production of compounds identical to those of the host by endophytic plant bacteria or microorganisms regulating host metabolism through other mechanisms (Khan et al., 2019).

## 5 Conclusion

In conclusion, we employed high-throughput sequencing and UPLC-Q-TOF-MS to investigate the rhizosphere microorganisms and metabolites of *D. denneanum* from different cultivation modes in the same environment We observed metabolites among the different cultivation modes. Moreover, the cultivation modes also influenced the composition of both rhizobacterial and fungal communities. Interestingly, the composition of endophytic fungal communities exhibited a greater resemblance to the rhizospheric fungal communities. This phenomenon may be associated with the characteristics inherent to orchidaceae plants. Furthermore, our subsequent analyses revealed that the rhizosphere microbial community influences the synthesis of plant metabolites, notably linking the biosynthesis of the pharmacologically active compound gigantol to the presence of *Occallatibacter* and *Clonostachys*. This study contributes to the literature on *Dendrobium* spp. root microorganisms provides a scientific basis for the rational cultivation of *Dendrobium* spp., and provides ideas for the study of mycorrhizal fungi in orchids.

## Data availability statement

The datasets presented in this study can be found in online repositories. The names of the repository/repositories and accession number(s) can be found below: https://www.ncbi.nlm.nih.gov/, PRJNA1009501.

## Author contributions

LC: Methodology, Project administration, Visualization, Writing – original draft, Writing – review & editing. HD: Methodology, Project administration, Visualization, Writing – original draft, Writing – review & editing. XC: Methodology, Visualization, Writing – original draft. JW: Visualization, Writing – original draft. YH: Validation, Writing – review & editing. HC: Validation, Writing – review & editing. YL: Funding acquisition, Project administration, Validation, Writing – review & editing.

## Supplementary material

Supplementary Figure 1 Ion flow diagram of metabolites of *D. denneanum*.

Supplementary Figure 2 The bacterial and fungi taxa with their LDA scores. Based on LEfSe results, the taxa were ranked according to their LDA scores. **(A)** rhizosphere bacteria, **(B)** root endosphere bacteria, **(C)** rhizosphere fungi, **(D)** root endosphere fungi.
