# Supplementary figures and images for: Cultivation modes impacting root microbiomes and metabolites in medicinal orchid Dendrobium denneanum
Source: Front Microbiomes. 2023 Dec 20;2:1287336. doi: 10.3389/frmbi.2023.1287336 (PMC12993561; doi:10.3389/frmbi.2023.1287336)

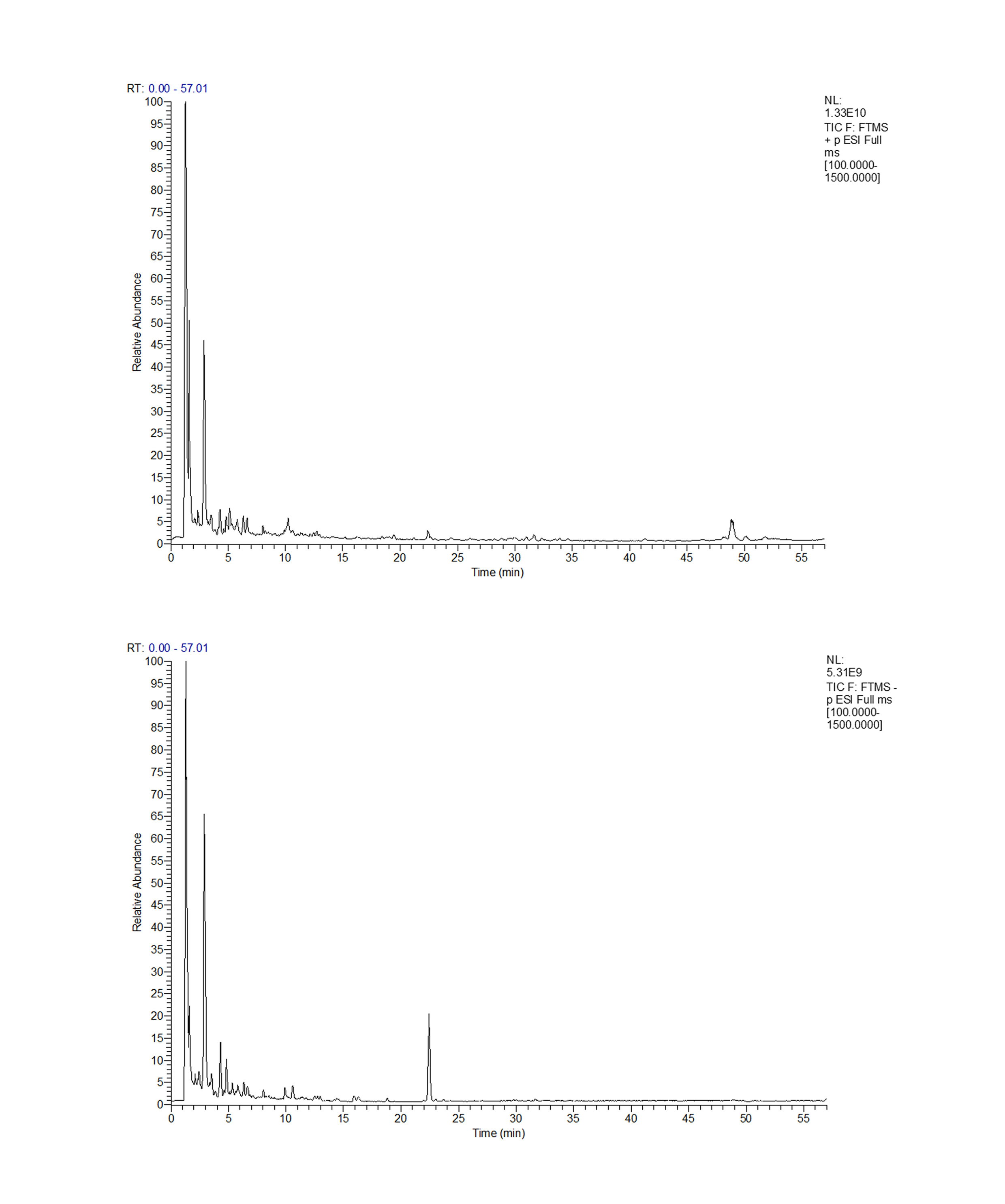

Supplement: Supplementary Figure 1 — Ion flow diagram of metabolites of D. denneanum. [file Image_1.jpeg]

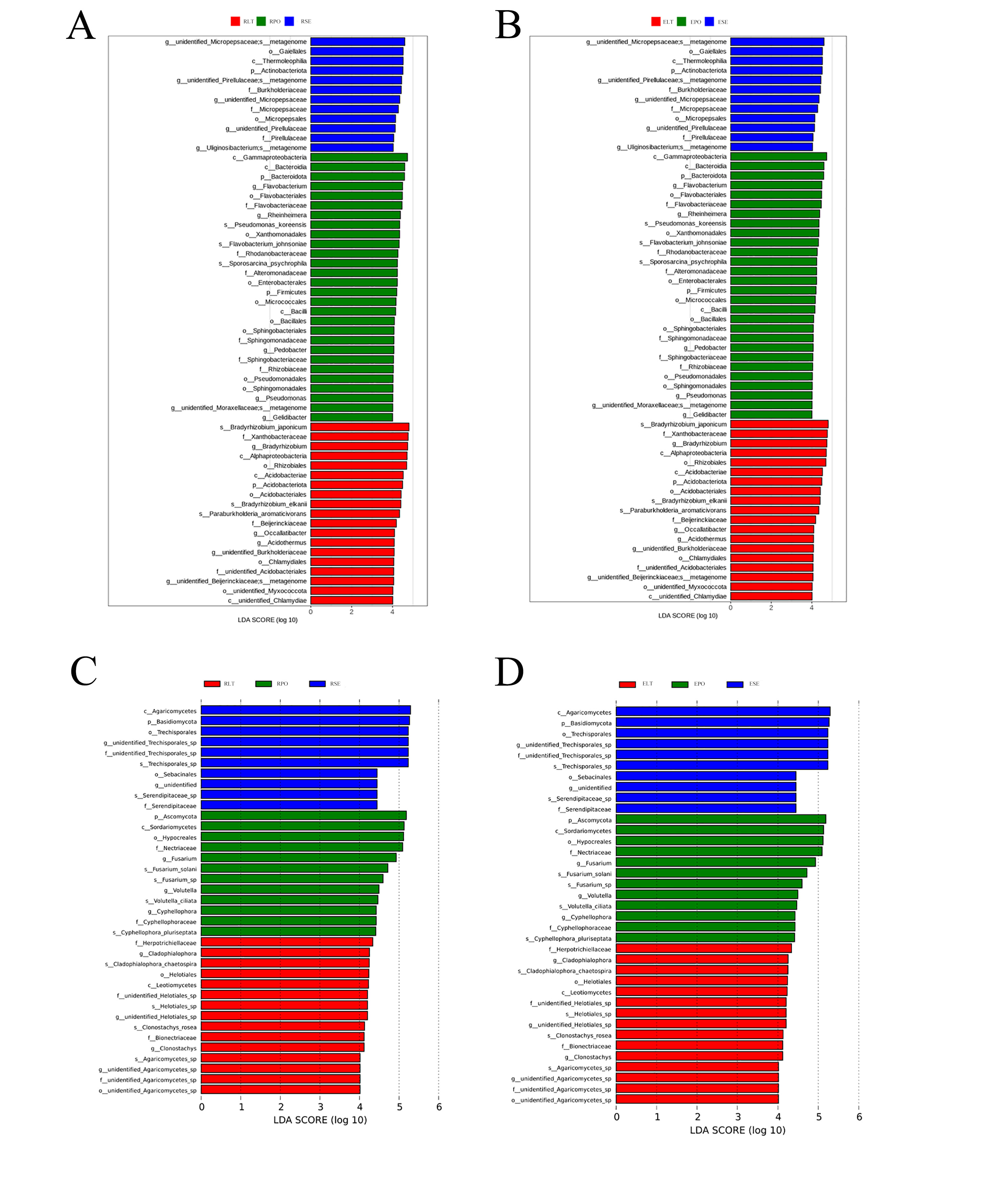

Supplement: Supplementary Figure 2 — The bacterial and fungi taxa with their LDA scores. Based on LEfSe results, the taxa were ranked according to their LDA scores. (A) rhizosphere bacteria, (B) root endosphere bacteria, (C) rhizosphere fungi, (D) root endosphere fungi. [file Image_2.jpeg]
